# Nucleosome Acidic Patch Promotes RNF168- and RING1B/BMI1-Dependent H2AX and H2A Ubiquitination and DNA Damage Signaling

**DOI:** 10.1371/journal.pgen.1004178

**Published:** 2014-03-06

**Authors:** Justin W. Leung, Poonam Agarwal, Marella D. Canny, Fade Gong, Aaron D. Robison, Ilya J. Finkelstein, Daniel Durocher, Kyle M. Miller

**Affiliations:** 1 Department of Molecular Biosciences, University of Texas at Austin, Austin, Texas, United States of America; 2 Institute for Cellular and Molecular Biology, University of Texas at Austin, Austin, Texas, United States of America; 3 Samuel Lunenfeld Research Institute, Mount Sinai Hospital, Toronto, Ontario, Canada; University of Washington, United States of America

## Abstract

Histone ubiquitinations are critical for the activation of the DNA damage response (DDR). In particular, RNF168 and RING1B/BMI1 function in the DDR by ubiquitinating H2A/H2AX on Lys-13/15 and Lys-118/119, respectively. However, it remains to be defined how the ubiquitin pathway engages chromatin to provide regulation of ubiquitin targeting of specific histone residues. Here we identify the nucleosome acid patch as a critical chromatin mediator of H2A/H2AX ubiquitination (ub). The acidic patch is required for RNF168- and RING1B/BMI1-dependent H2A/H2AXub *in vivo*. The acidic patch functions within the nucleosome as nucleosomes containing a mutated acidic patch exhibit defective H2A/H2AXub by RNF168 and RING1B/BMI1 *in vitro*. Furthermore, direct perturbation of the nucleosome acidic patch *in vivo* by the expression of an engineered acidic patch interacting viral peptide, LANA, results in defective H2AXub and RNF168-dependent DNA damage responses including 53BP1 and BRCA1 recruitment to DNA damage. The acidic patch therefore is a critical nucleosome feature that may serve as a scaffold to integrate multiple ubiquitin signals on chromatin to compose selective ubiquitinations on histones for DNA damage signaling.

## Author Summary

Post-translational modifications of histones play important roles in regulating both the structure and function of chromatin. As all DNA based processes, including transcription, DNA replication and DNA repair, occur within the context of chromatin, the actual *in vivo* substrate of these reactions is chromatin. Thus, understanding these processes within the context of chromatin is vital for providing mechanistic insights into chromatin-based processes, including DNA damage signaling and genome maintenance. Here we identify a structure within H2A and H2AX termed the acidic patch that promotes the activity of two independent ubiquitin E3 ligase complexes, RNF168 and RING1B/BMI1, and is required for DNA damage ubiquitin signaling. We show directly *in vitro* and *in vivo* that this nucleosome structure is critical for histone H2A and H2AX ubiquitinations and the DNA damage response in cells. In addition, we engineered a novel biological tool that blocked the nucleosome acidic patch of all histone H2A species leading to the repression of the DNA damage response in cells. Collectively, DNA damage factors elicit their response not only through histone modifications such as ubiquitin but also through interactions within nucleosome surface structures to activate DNA damage signaling.

## Introduction

Eukaryotic DNA is bound by histone proteins and organized into chromatin, the true *in vivo* substrate of transcription, replication and DNA repair, processes that are important in preserving genome integrity. Chromatin structure and function are highly regulated by histone post-translational modifications (PTMs) [1]. Histones are modified on distinct amino acid residues by different PTMs, such as phosphorylation, acetylation and ubiquitination, including several that are involved in DSB repair [2]. Upon DSB formation, H2AX is phosphorylated on Ser-139 within its C-terminal tail by the PIKK family kinases ATM, ATR and DNA-PK, to yield γH2AX [3]. γH2AX can be generated over a megabase of chromatin surrounding DSBs, thus creating microscopically-visible ionizing radiation-induced nuclear foci (IRIF) [4], [5]. γH2AX creates a binding site for the DNA damage protein MDC1, which promotes the localization of other DNA damage factors to damage sites [2]. Numerous E3 ubiquitin ligases including RNF8, RNF168, BRCA1, RING1B and BMI1 are recruited to DNA lesions [6], [7]. Collectively these DNA damage factors orchestrate the DNA damage response (DDR) that is a complex signaling network that is critical in regulating DNA damage signaling and repair [6], [8], [9]. Ubiquitin-mediated responses to DNA damage include histone H2A and variant H2AX ubiquitinations (H2A/H2AXub). Indeed, H2A/H2AX is ubiquitinated by RNF168, which targets Lys-13/15 within the N-terminal tail [10]–[12], and RING1B/BMI1 that ubiquitinates C-terminal Lys-118/119 of H2A/H2AX [13]–[16]. Ubiquitinated histones H2AX and H2A mediate the chromatin association of both the mediator protein 53BP1 and the repair factor BRCA1. These interactions occur through binding to Ubiquitin-interaction motif (UIM) domains in 53BP1 and in the BRCA1-interacting protein RAP80 [17], [18]. Thus, site-specific histone ubiquitinations mediate critical signaling events that promote sensing and repair of DNA damage in mammalian cells [2], [6], [19]. Although the role of histone ubiquitination is well established in DNA damage signaling, it is unclear how the ubiquitin E3 ligases recognize their specific lysine targets on histones within the context of the nucleosome. Whether the nucleosome itself is involved in mediating the site-specific ubiquitin modifications on histones in response to DNA damage or other biological signals involving histone ubiquitinations has not yet been established. In this study, we find that the nucleosome acidic patch is required for RNF168- and RING1B/BMI1-dependent H2A and H2AX ubiquitination.

## Results/Discussion

### The acidic patch promotes H2AX/H2A ubiquitination

Ubiquitination of histones has emerged as a critical component of the DNA damage signaling pathway in mammalian cells [6]. We previously identified several mutations that reduced H2AX ubiquitin levels in undamaged cells [20]. One such mutation, H2AX-E92A resided in the acidic patch region of the nucleosome. Expression of tagged versions of human H2AX and H2A in human HEK293T cells revealed a full-length protein species of predicted size as well as a slower migrating ubiquitinated form for both human H2AX and H2A (Figure S1A, B). Mutation of glutamic acid 92 to alanine (E92A) reduced H2AX and H2A ubiquitination (H2AX/H2Aub, Figure S1A, B). These results identify the amino acid E92 of human H2AX/H2A as an important residue for H2AX/H2Aub.

**Figure 1:**
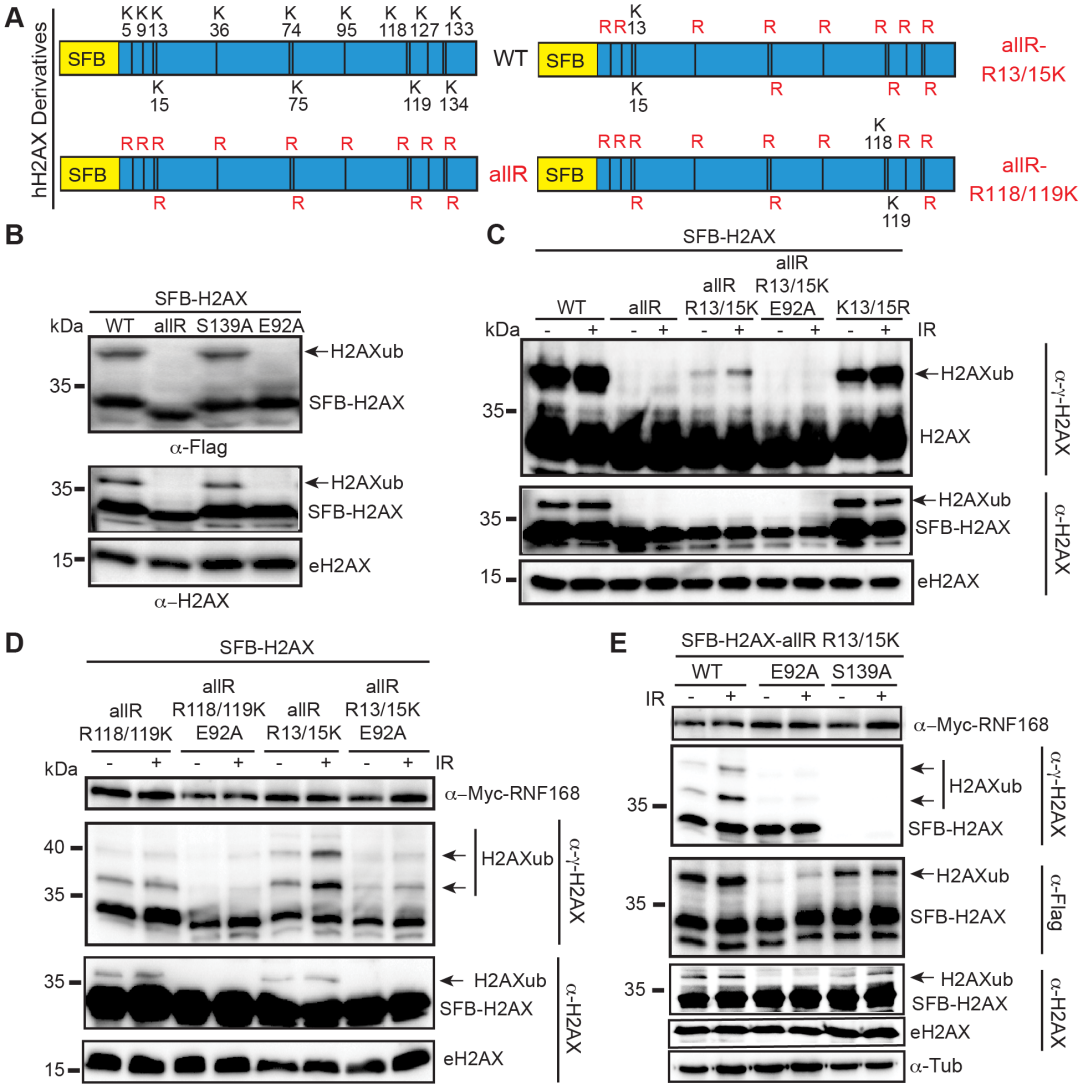
Mutation of the acidic patch impairs human H2AX and H2A ubiquitination. (**A**) Schematic of all H2AX lysines (K) and mutant derivatives. allR represents an all lysine (K) to arginine (R) version of H2AX. Additional site-specific reversions from arginine to lysine within the allR H2AX derivate are indicated. (**B**) H2AX-allR and acidic patch mutation E92A reduces H2AXub. WT or E92A H2AX/H2A constructs were transfected into HEK293T cells and analyzed by western blotting with the indicated antibodies. Arrows indicate ub forms. (SFB = S-tag, Flag epitope tag, and streptavidin-binding peptide tag; e = endogenous). Molecular weights (kDa) are indicated on the left of each panel. HEK293T cells were used for all cellular assays. (**C**) H2AX-K13/15 dependent ubiquitination requires the acidic patch. H2AX and derivatives were expressed in HEK293T cells (−) or (+) ionizing radiation (IR, 20 Gy). Samples were analyzed as in A 6 h post-IR treatment. (**D**) H2AX-K13/15 and K118/119-dependent ubiquitination requires the acidic patch. Cells were co-transfected with H2AX and derivatives along with Myc-RNF168 and analyzed as in C. (**E**) Phospho-competent H2AX S139 is not required *in cis* for H2AX K13/15ub. Cells were analyzed as in C. tub = tubulin loading control.

We next sought to define the contribution of the acidic patch region of the nucleosome towards H2AX/H2Aub and the DDR. H2AX/H2A is specifically ubiquitinated on the N-terminal Lys-13/15 by RNF168 [10]–[12], as well as on the C-terminal Lys-118/119 by RING1B/BMI1 [13]–[16]. Therefore, an important question was to determine which sites on H2AX rely on the acidic patch for ubiquitination. To answer this question, we first created a lysine-free human H2AX where all lysine residues were mutated to arginines. These mutations maintain the basic charge at each amino acid location but are unable to be ubiquitinated (Figure 1A). As expected, expression of H2AX-allR in HEK293T cells confirmed that this mutant lacked any detectable ubiquitination, similarly to H2AX-E92A (Figure 1B). Unlike these H2AX derivatives, mutation of the DNA damage induced phosphorylation site on H2AX (S139) to an unphosphorylatable residue (S139A) did not affect H2AXub (Figure 1B). Having identified a mutant H2AX derivative that lacked ubiquitination, we then reverted specific arginine residues in this mutant back to lysine residues that are contained in WT H2AX (Figure 1A). This strategy allowed us to unambiguously identify site-specific ubiquitinations within H2AX. As expected, H2AX mutants lacking K118/119 exhibited a large reduction in mono-ubiquitination (Figure 1C). This confirmed previous work showing that these sites on H2AX/H2A are the major lysine acceptor sites for mono-ubiquitination [2], [21]. Interestingly, we observed ubiquitination of the H2AX derivative containing only K13/15 as acceptor sites for ubiquitin (Figure 1C). We also observed an increase in K13/15ub on this H2AX derivative upon DNA damage, which is consistent with previous studies showing that a small fraction of H2AX becomes ubiquitinated on K13/K15 following DNA damage by the E3 ubiquitin ligase RNF168 [10]–[12]. To assess the contribution of the acidic patch towards H2AX K13/15ub, we tested whether an E92A mutation would affect H2AX K13/15ub. Combining the E92A mutation within the H2AX derivative that could only be ubiquitinated on K13/15 abolished any detectable ubiquitination at these sites within H2AX (Figure 1C). We next tested whether the acidic patch also affected the ubiquitination of K118/119 of H2AX. Analysis of an H2AX derivative that could only be ubiquitinated on K118/119 showed that this protein was readily ubiquitinated and mutation of the acidic patch diminished H2AXub at these specific lysine sites (Figure S2).

RNF168 is a limiting factor within the DDR and overexpression of RNF168 increases H2AX-K13/15ub levels but not H2AX-K118/119ub levels [10]–[12], [22]. In agreement with these studies, we observed that overexpression of RNF168 increased H2AX-K13/15ub but not H2AX-K118/119ub (Figure 1D, S2). In accordance with our results from Figure 1C, mutation of the acidic patch decreased H2AX-K13/15ub levels, even under conditions where RNF168 is overexpressed and not limiting (Figure 1D). H2AX-K13/15ub is mediated by RNF168 whose recruitment to sites of DNA damage requires MDC1 and RNF8 [23], [24], which in turn require H2AX phosphorylation on S139 [24]–[27]. Collectively, these findings suggest that γH2AX may be required for H2AX-K13/15ub. To test this possibility, we mutated S139 within the H2AX-allR-R13/15K derivative to monitor specifically H2AX-K13/15ub in either the presence or absence of S139. While E92A abolished H2AX-K13/15ub, the S139A mutation did not affect ubiquitination at these sites (Figure 1E). These results show that S139 is not required *in cis* for H2AX-K13/15ub under these conditions. We note that these experiments were performed in cells containing WT H2AX that could provide functional residues *in trans* for H2AX-K13/15ub. These experiments were done in the presence of overexpressed RNF168, which could bypass the requirement for S139 phosphorylation for its recruitment to chromatin. In overexpression conditions, RNF168 accumulates at sites of endogenous DNA damage marked by 53BP1 [22], which we note requires K13/15ub on H2A/H2AX [10]. Regardless, under either limiting or non-limiting conditions for RNF168, we find that the acidic patch is required for H2AX-K13/15ub (Figure 1C–E).

### Nucleosome acidic patch is required for H2AX/H2Aub *in vitro*

**Figure 2:**
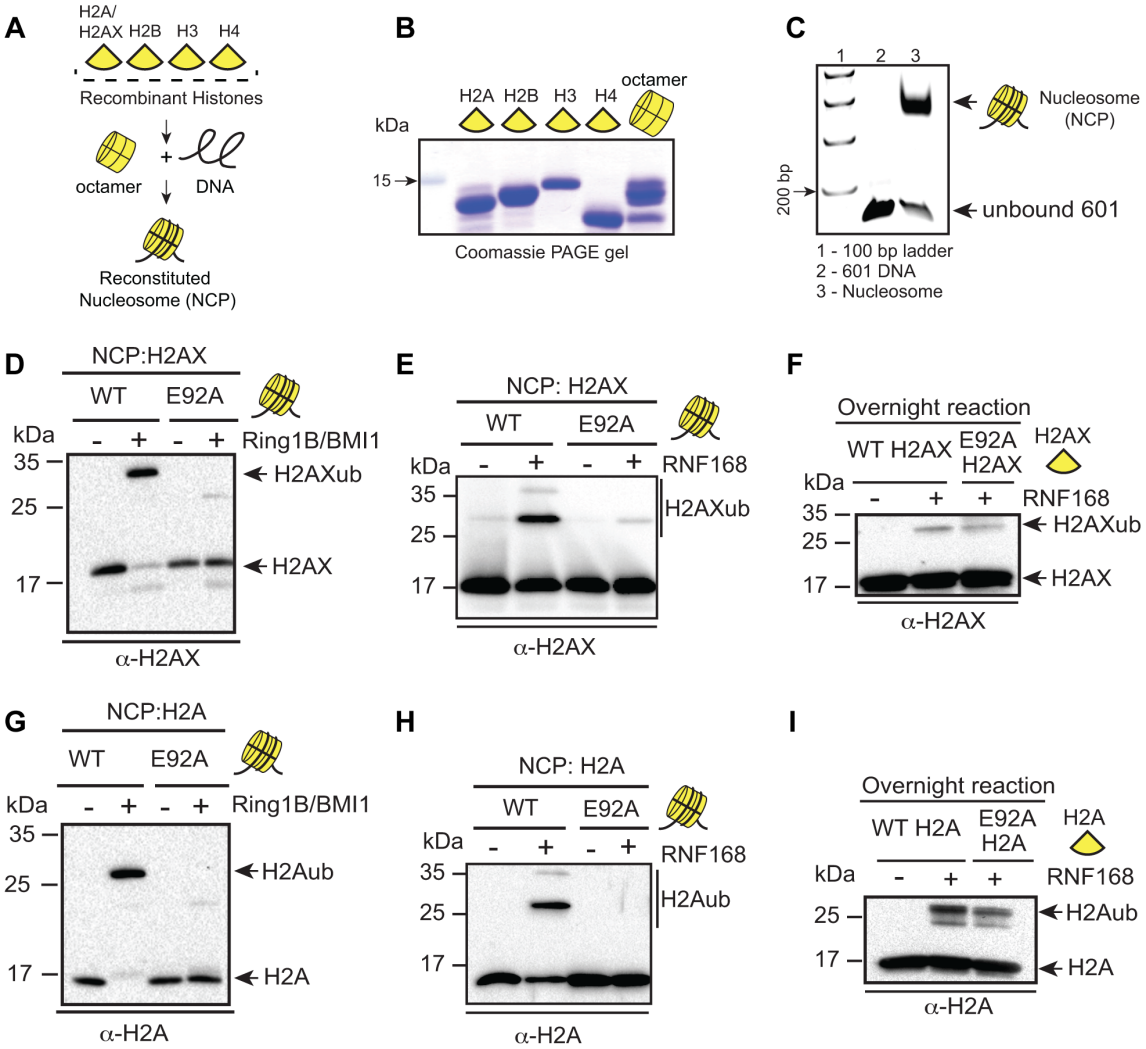
RING1B/BMI1- and RNF168-dependent ubiquitination of H2AX/H2A requires the nucleosome acidic patch *in vitro*. (**A**) Schematic for *in vitro* reconstitution of nucleosome core particles (NCPs). (**B**) Bacterially expressed and purified human histones. Histones were expressed, purified and reconstituted as described in methods. (**C**) Analysis of *in vitro* reconstituted NCPs. The 147 bp 601 DNA fragment was analyzed alone or after NCP reconstitution. DNA ladder indicates size (bp). (**D and E**) RING1B/BMI1 and RNF168 readily ubiquitinate H2AX within WT NCPs but not NCPs containing a mutation in the acidic patch (H2AX-E92A). In vitro Ub assays (4 h) were performed as described in methods. (**F**) RNF168 ubiquitinates WT H2AX and H2AX-E92A similarly when assayed in the context of free histones. Assays were performed as in E except with free histones and reactions were performed overnight. (**G and H**) RING1B/BMI1 and RNF168 readily ubiquitinate H2A within WT NCPs but not NCPs containing a mutation in the acidic patch (H2A-E92A). Experiments were performed as in D and E using H2A. (**I**) RNF168 ubiquitinates free WT H2A and H2A-E92A similarly. Experiments performed as in F.

Our results strongly suggested that the acidic patch is required for both K13/15 and K118/119 H2AX/H2Aub. We next sought to test whether the effect of the acidic patch mutation on H2AX/H2Aub was direct, as well as to analyze the role of the acidic patch in mediating site-specific ubiquitinations with their associated E3 ligases. To assess these questions, we reconstituted H2AX and H2A nucleosome core particles (NCPs, Figure 2A–C) with or without the acidic patch mutation (i.e. E92A) and subjected them to *in vitro* ubiquitination (Ub) assays. Previous studies have established that bacterially expressed and purified RNF168 and RING1B/BMI1 complexes catalyze the specific addition of ubiquitin on H2AX/H2A NCPs at K13/15 and K118/119 respectively [10], [11]. Using the same constructs and experimental conditions, we performed *in vitro* Ub assays with H2AX NCPs with or without the E92A acidic patch mutation. As expected, RING1B/BMI1 and RNF168 ubiquitinated H2AX within WT NCPs (Figure 2D, E). In contrast, both E3 ligase complexes were unable to efficiently ubiquitinate NCPs containing E92A mutation in H2AX (Figure 2D, E). These effects appear to occur within the context of the nucleosome as RNF168 could ubiquitinate the free form of H2AX whether it was WT or contained the acidic patch E92A mutation (Figure 2F). We performed identical experiments with H2A WT and E92A NCPs and obtained the same results (Figure 2G–I). As another control, we subjected H2AX and H2A WT and E92A NCPs to *in vitro* methylation assays with SET8, a methyltransferase that is active only within the context of the nucleosome for methylating H4K20 [28]. The acidic patch mutation did not affect nucleosome specific SET8 methylation suggesting the E92A mutation does not overtly disorder the NCP (Figure S3). These *in vitro* results are consistent with our *in vivo* data and demonstrate that RNF168 and RING1B/BMI1 require the nucleosome acidic patch of H2AX/H2A to promote site-specific ubiquitination of H2AX/H2A K13/15 and H2AX/H2A K118/119 respectively.

### Nucleosome acidic patch of H2AX and the DNA damage response

Our findings show that the nucleosome acidic patch mediates both H2AX/H2A K13/15ub by RNF168 and H2AX/H2A K118/119ub by RING1B/BMI1. Several studies have shown that RING1B/BMI1 participates in the DDR although a clear function for H2AX/H2A K118/119ub is as yet unidentified [13]–[16], [29]–[31]. In contrast, the function of H2AX/H2A K13/15ub by RNF168 was recently elucidated and is well defined [10]. Indeed, RNF168-dependent H2AX/H2A K15ub is selectively recognized by the ubiquitination-dependent recruitment motif (UDR) of 53BP1 that, together with its Tudor domain, reads a bivalent ubiquitin-methylation signal at DNA damage sites to recruit the DDR factor 53BP1. A clear prediction of this mechanism is that 53BP1 recruitment to sites of DNA damage would be perturbed in the absence of H2AX-K13/15ub and/or H2A K13/15ub. We chose to next focus on the role of the acidic patch in regards to RNF168-dependent H2AX/H2A K13/15ub *in vivo* since we could utilize 53BP1 foci formation as an *in vivo* read-out for functional H2AX/H2A-K13/15ub.

**Figure 3:**
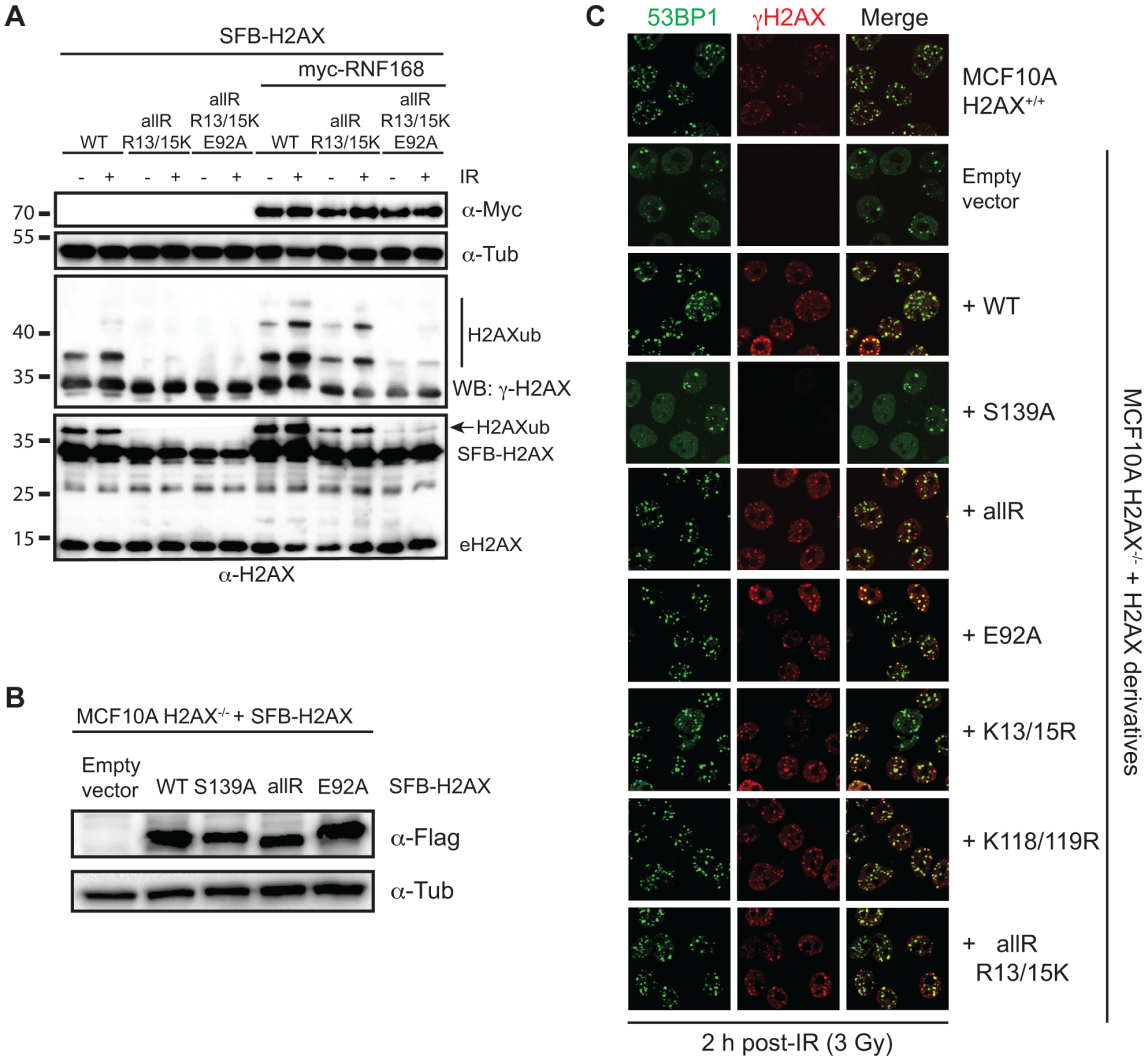
Regulation of H2AX-K13/K15ub by RNF168 requires the acidic patch. (**A**) Maximum H2AX-K13/15ub levels (−) or (+) RNF168 is dependent on the acidic patch. HEK293T cells were transfected with H2AX and derivatives either (−) or (+) RNF168 and analyzed as in Figure 1D. (**B**) Human H2AX and derivatives reconstituted in MCF10A H2AX^−/−^ cells. Western blot analysis of the indicated MCF10A H2AX^−/−^ stable cell lines. (**C**) H2AXub is dispensable for 53BP1 foci formation after DNA damage. Reconstituted MCF10A H2AX^−/−^ cells stably expressing H2AX and H2AX mutants were analyzed by immunofluorescence (IF) with the indicated antibodies. Cells were treated with 3 Gy IR and analyzed by IF 2 h post-IR.

Because RNF168 specifically targets H2AX-K13/15, we sought to characterize further our H2AX derivatives where K13/15 are the only lysines available for ubiquitination and to ascertain the contribution of both the acidic patch and RNF168 expression levels on H2AX-K13/15 ubiquitin levels. Expression of SFB-tagged WT H2AX resulted in clearly identifiable mono-ubiquitinated species whose electrophoretic mobility was retarded as expected due to the presence of a 9 kDa ubiquitin protein (Figure 3A). Rendering WT H2AX unmodifiable by ubiquitin on all but K13/15 resulted in an almost complete loss of mono-ubiquitinated H2AX (Figure 3A). This reduction was also observed when the acidic mutation E92A was added to this H2AX derivative. To analyze the contribution of both RNF168 and the acidic patch on H2AX-K13/15ub, we repeated these experiments in the presence of overexpressed Myc-tagged RNF168. Although we still observed reduced H2AXub in K13/15 only H2AX derivatives compared to WT H2AX, we now were able to specifically detect H2AX-K13/15ub using this H2AX derivative that only contained K13/15 (Figure 3A). Interestingly, we were able to detect a small increase in H2AX-K13/15ub upon DNA damage suggesting that this H2AX derivative was functioning within the DDR in cells (Figure 3A). Under these optimized conditions for specifically detecting H2AX-K13/15ub, mutation of the acidic patch (i.e. E92A) resulted in a large reduction in H2AX-K13/15ub levels either in the presence or absence of DNA damage (Figure 3A). Thus, we could detect DNA damaged induced H2AX-K13/15ub in the presence of RNF168, and in all conditions tested, H2AX-K13/15ub required the acidic patch.

Having now characterized H2AX derivatives for their ubiquitination on K13/15, K118/119 or in the absence of lysines, we sought to determine whether H2AX ubiquitinations were required *in vivo* for the DDR and more specifically for 53BP1 foci formation. Up to now, all of our experiments analyzing H2AX derivatives were performed in the presence of WT H2AX. To overcome this limitation, we turned to a human cell line deleted for H2AX, MCF10A H2AX^−/−^, that we previously characterized [20]. To test the contribution of H2AXub for 53BP1 IRIF, we stably reconstituted MCF10A H2AX^−/−^ with WT H2AX and derivatives to compare the ability of site-specific mutations in ubiquitinated sites on H2AX to complement the defect of 53BP1 IRIF that occurs in these cells in the absence of H2AX. We first created stable cell lines expressing H2AX constructs to be tested and selected clones for each that expressed H2AX in the majority of cells and to similar protein levels as the WT H2AX reconstituted cell line (Figure 3B, S4 and data not shown). To assess 53BP1 IRIF, we analyzed several H2AX derivatives for their ability to rescue defective 53BP1 IRIF in MCF10A cells lacking H2AX. As we previously reported, MCF10A H2AX^−/−^ and MCF10A H2AX^−/−^+H2AX S139A are unable to support equivalent recruitment of 53BP1 into IRIF compared to WT MCF10A cells (Figure 3C). Surprisingly, all H2AX derivatives tested, including a lysine-less H2AX (allR) that cannot support ubiquitination on either K13/15 or K118/119, were able to fully support 53BP1 IRIF (Figure 3C). Thus, although S139 phosphorylation is required for 53BP1 IRIF in these cells, H2AXub (including K13/15 or K118/119), as well as the H2AX acidic patch, is dispensable for 53BP1 IRIF (Figure 3C). As DNA damage dependent H2A-K13/15ub also occurs, these results suggest that γH2AX could function *in trans* to promote H2A- K13/15ub that would be sufficient to mediate 53BP1 recruitment to sites of DNA damage. One hypothesis could be that DNA damage induced H2AX phosphorylation on S139 could mediate an initial ubiquitination on H2AX-K13/15 that would be required to amplify RNF168-dependent H2Aub. Similarly, the nucleosome acidic patch of H2AX could initiate the recruitment and activation of RNF168 that would in turn trigger the start of this ubiquitin-dependent signaling pathway. However, our results argue against these hypotheses and instead suggest that the acidic patch of H2A, as well as H2Aub, can compensate for H2AXub in the DDR to support 53BP1 IRIF. Testing the role of the H2A acidic patch and H2Aub *in vivo* is extremely challenging due to the unavailability of a mutation system for H2A in human cells. Regardless, our findings establish that the acidic patch of H2AX, as well as H2AXub, is dispensable for 53BP1 IRIF in human cells.

### Nucleosome acidic patch is required for the DDR *in vivo*

To overcome the limitations of studying histone mutants *in vivo* and to validate the requirement of the nucleosome acidic patch in promoting H2AX/H2Aub and subsequent DDR signaling, we sought to identify an experimental approach to target the acidic patch regions of both H2A and H2AX *in vivo*. The nucleosome acidic patch of H2A has been shown to interact with several proteins including histone H4, the Kaposi's sarcoma–associated herpesvirus (KSHV) protein LANA, IL-33, HMGN2 and RCC1 [32]–[36]. The finding that several proteins interact through this nucleosome region has suggested that the nucleosome acidic patch acts as a “chromatin platform” to mediate various cellular signals via their interactions with chromatin through the acidic patch. As our data has identified the nucleosome acidic patch of H2AX and H2A as a requirement for RNF168- and RING1B/BMI1-dependent H2AX/H2Aub *in vitro* and *in vivo*, we set out to test whether expression of a known acidic patch interacting protein could interfere with these DDR factors. This experimental approach has the advantage of blocking both H2A and H2AX acidic patch regions, a potential necessity for uncovering the function of this nucleosome domain in the DDR. Results from these experiments would further define the role of the nucleosome acidic patch of both H2A and H2AX in the DDR and would allow us to test our hypothesis that the acidic patch of H2A and H2AX functions in the DDR *in vivo*, at least in part by promoting H2AX/H2A-K13/15ub.

**Figure 4:**
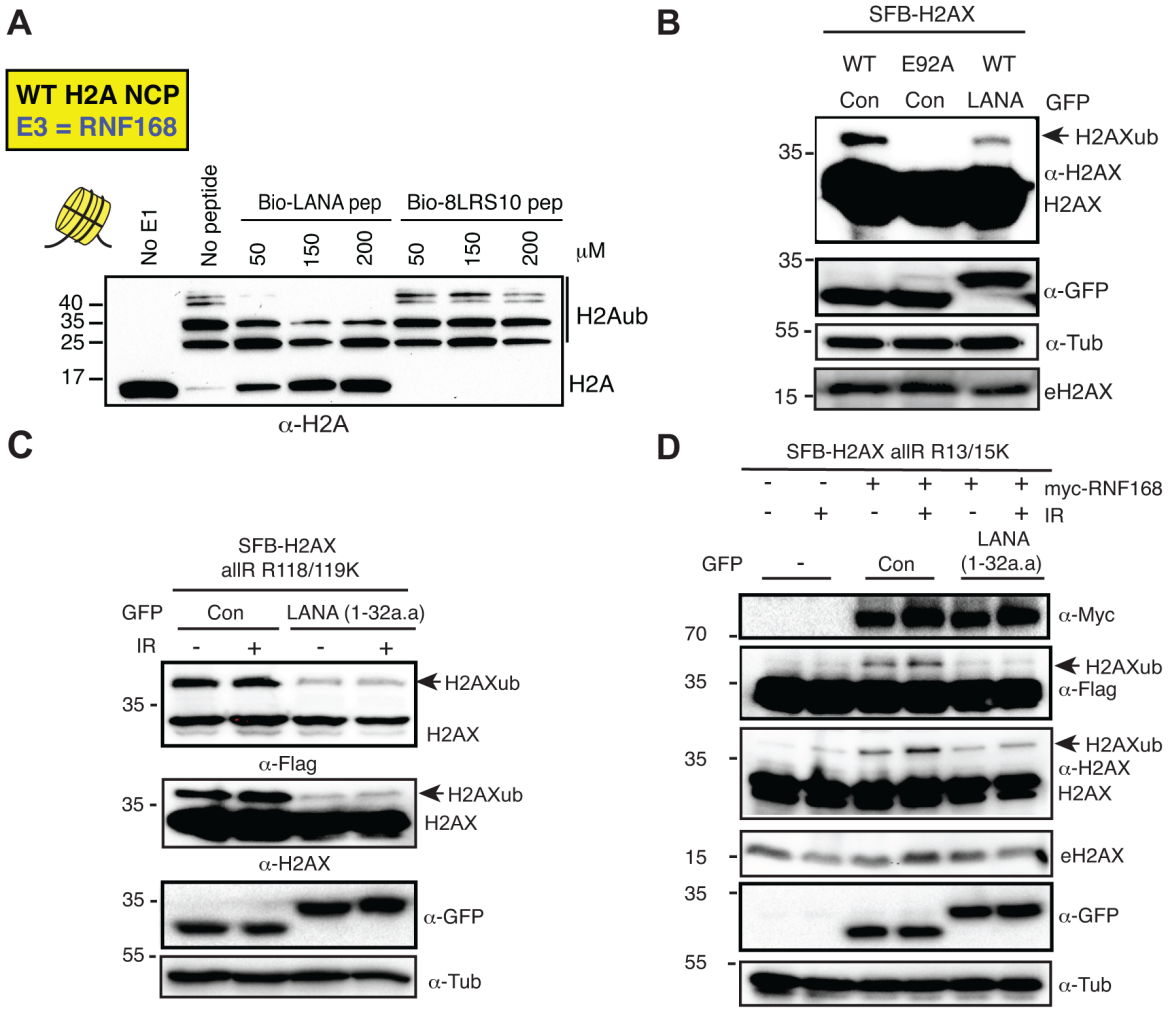
The KSHV LANA peptide inhibits histone ubiquitination *in vitro* and *in vivo*. (**A**) The acidic patch interaction region of LANA inhibits RNF168-dependent H2Aub *in vitro*. *In vitro* Ub assays were performed (−) or (+) either LANA peptide or a mutant LANA peptide (8LRS10) that does not interact with the nucleosome acidic patch. Assays were performed as in Figure 2 with increasing concentrations of peptides (µM) as indicated (4 h reactions). (**B**) Expression of GFP-LANA (1–32a.a.) reduces H2AXub. HEK293T cells were transfected with the indicated constructs and analyzed by western blotting as in Figure 1B. (**C**) GFP-LANA (1–32a.a.) reduces H2AXub at K118/119. Experiments were performed as in B using H2AX-allR-R118/119K with or without IR treatment. (**D**) Expression of GFP-LANA (1–32a.a.) reduces RNF168-dependent H2AX-K13/15ub. Experiments were performed and analyzed as in Figure 3A with the indicated constructs, with or without IR. Arrows indicate H2AXub protein species. e = endogenous; con = control GFP alone.

The KSHV latency-associated nuclear antigen (LANA) interacts with the nucleosome acidic patch of H2A to tether episomes to chromosomes [32]. The first 32 amino acids of LANA comprise the acidic patch interacting region and expression of a GFP fusion with this minimal region in cells is sufficient to target this small truncated region of the protein to mitotic chromosomes [32]. Additionally, mutation of the 8–10 amino acid region (named 8LRS10) of this 32 amino acid LANA peptide abolishes the interaction of LANA with the nucleosome acidic patch. To assess whether this acidic patch interacting peptide from LANA could compete with RNF168- and RING1B/BMI1-dependent H2AX/H2Aub, we synthesized the minimal acidic patch interacting peptide from LANA along with the 8LRS10 mutant peptide and analyzed the effects of these peptides on our previously characterized *in vitro* Ub assays. Interestingly, the acidic patch binding LANA peptide reduced H2Aub that was catalyzed by both RING1B/BMI1 and RNF168 in a concentration-dependent manner (Figure 4A, S5). The reduction of H2Aub by the LANA peptide required the ability to bind the acidic patch as the 8LRS10 mutant peptide was unable to compete away H2Aub. These results supported our previous findings that the acidic patch was directly promoting histone ubiquitination by these E3 ligases and also suggested that the LANA peptide could interfere with this reaction in cells.

To begin to address this question, we wanted to ask whether we could observe a decrease in H2AXub in cells expressing LANA peptide. We cloned and engineered a GFP-fusion of LANA containing only the first 32 amino acids (GFP-LANA (1–32a.a.), [32]). Next, we co-transfected our H2AX derivatives with GFP-LANA and analyzed H2AXub by western blotting. We observed that the ubiquitination of WT H2AX, H2AX-K118/119 only and H2AX-K13/15 only were reduced when co-expressed with GFP-LANA in cells (Figure 4B–D). These results are in agreement with both our *in vitro* and *in vivo* data demonstrating that the nucleosome acidic patch of H2AX is required for K13/15 and K118/119 ubiquitination (Figure 1, 2).

**Figure 5:**
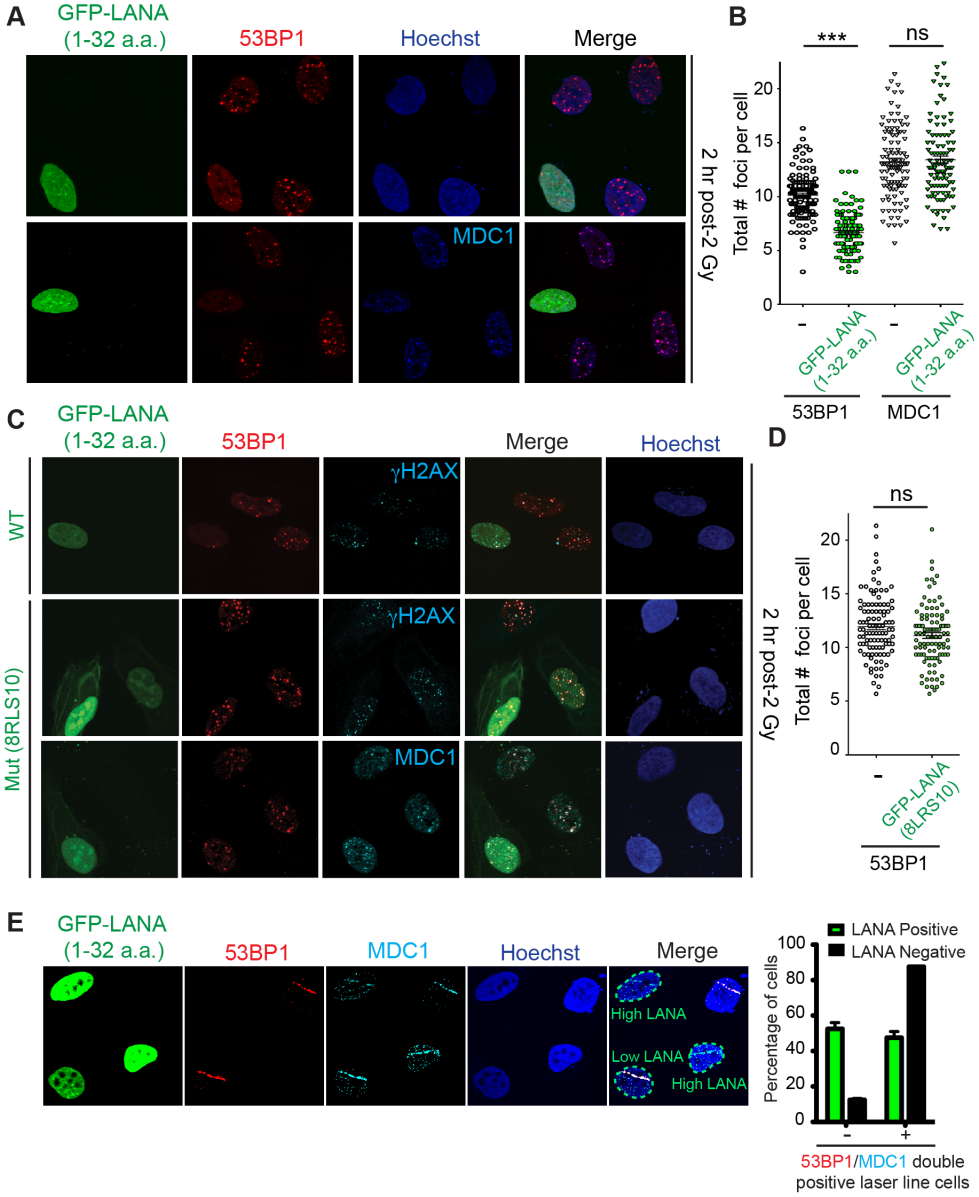
The nucleosome acidic patch is required *in vivo* for the DDR in human cells. (**A and B**) *In vivo* expression of the acidic patch interacting portion of LANA (1–32 amino acids) reduces 53BP1, but not MDC1, IRIF (ionizing radiation induced-foci). Human U2OS cells were transfected with GFP-LANA (1–32a.a.) followed by 2 Gy IR-treatment. Cells were analyzed by IF with the indicated antibodies 2 h post-IR. Representative IF images are shown. Nuclear DNA was visualized by Hoechst 33342 staining. Quantification of A is shown in B. 53BP1 and MDC1 IRIF were counted and graphed for cells (−) or (+) GFP-LANA (1–32a.a.). N = 3, >100 cells analyzed/experiment, error bars = SEM. Student's t-tests (paired) were performed and results indicated. *** = p-value<0.001, ns = not significant (i.e. p-value>0.05). (**C**) IF analysis of DDR factor foci formation after IR treatment in GFP-LANA and mutant GFP-LANA-8LRS10 expressing cells. Cells were treated with 2 Gy IR and processed for IF 2 h post-IR. IF analysis was performed as in A. (**D**) Quantification of 53BP1 IRIF from C. Graph represents values obtained from two independent experiments where foci from >100 cells were scored for GFP-LANA-8LRS10 expressing cells and non-GFP expressing cells. Error bars = SEM. Statistical analysis was performed as in B. (E) GFP-LANA (1–32a.a.) impairs recruitment of 53BP1 to laser damage. U2OS cells were transfected with GFP-LANA (1–32a.a.) followed by laser micro-irradiation. Cells were fixed and stained with antibodies as indicated 2 h post-laser damage. Quantification of 53BP1 and MDC1 laser lines were obtained from >50 damaged cells from two independent experiments. Error bars = SEM.

The ability of LANA to inhibit H2AXub *in vivo* suggested that cells expressing LANA would exhibit impaired DNA damage signaling. If this were indeed the case, a clear prediction would be that cells expressing LANA would exhibit reduced 53BP1 IRIF due to H2AX/H2Aub inhibition from LANA blocking RNF168 through the acidic patch. To test this possibility, we expressed GFP-LANA in human U2OS and HEK293T cancer cells and analyzed 53BP1 IRIF with and without GFP-LANA. Upon DNA damage, we observed reduced 53BP1 IRIF in cells expressing GFP-LANA compared to GFP alone expressing cells (Figure 5A, B, S6). Importantly, the upstream DDR factor MDC1, as well as γH2AX, were unaffected by GFP-LANA expression (Figure 5A–C). This is consistent with RNF168 inhibition by LANA as RNF168 acts downstream of γH2AX and MDC1 [23], [37]. To rule out any potential cell cycle effects due to GFP-LANA expression, we analyzed the cell cycle of GFP-LANA expressing cells. Analysis of these cells using FACS, DNA labeling by hoechst and phospho-Histone H3 (S10) immunostaining, a histone mark specific for mitotic cells, did not reveal any detectable differences in cell cycle stage or DNA staining between control and GFP-LANA expressing cells (Figure S7A–D). In addition, expression of mutant GFP-LANA-8LRS10, a mutation that is unable to bind the acidic patch, had no discernable effect on 53BP1 IRIF showing that the effect of GFP-LANA on the DDR required its interaction with the nucleosome acidic patch (Figure 5C–D). We also confirmed the inhibition of 53BP1, but not MDC1, in GFP-LANA expressing cells by laser micro-irradiation (Figure 5E). We observed that cells expressing high levels of GFP-LANA were able to fully inhibit 53BP1 recruitment to laser damage compared to cells expressing lower levels of GFP-LANA (Figure 5E). These results are consistent with GFP-LANA targeting the nucleosome acidic patch resulting in inhibition of 53BP1 recruitment to DNA damage.

**Figure 6:**
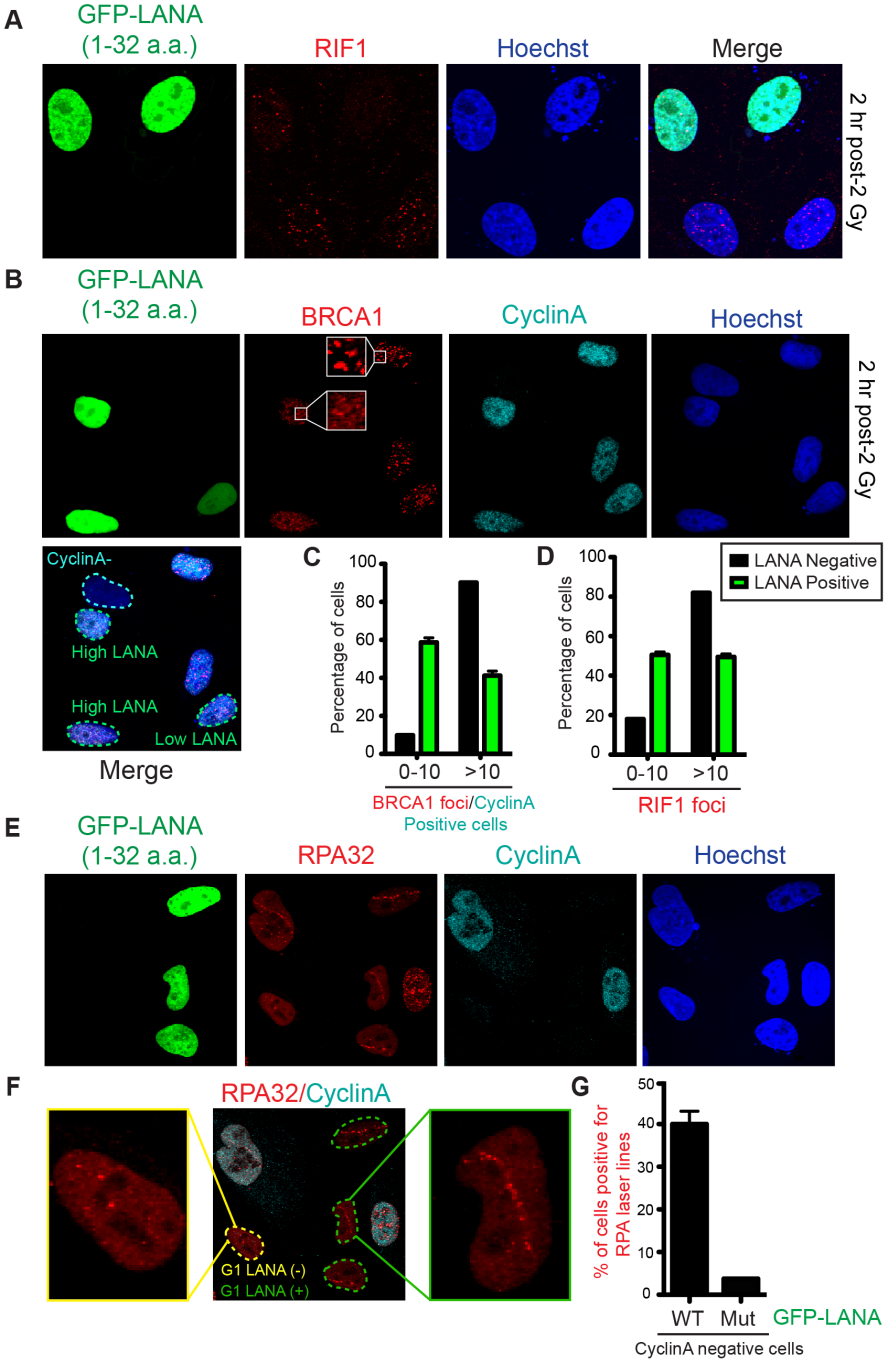
Nucleosome acidic patch promotes RNF168-dependent DDR signaling and inhibition of DNA resection in G1. (**A and B**) RIF1 and BRCA1 IRIF are impaired in GFP-LANA (1–32a.a.) expressing cells. Experiments were performed as in Figure 5A. Representative images are shown. CyclinA negative and high/low LANA expressing cells are indicated in the merged image. Note: CyclinA marks S/G2 cells. (**C and D**) Quantification of RIF1 and BRCA1/CyclinA-positive IRIF from A and B. Graphs represent values obtained from two independent experiments where foci from >100 cells were quantified. Error bars = SEM. (**E**) GFP-LANA (1–32a.a.) expressing cells exhibit DNA end-resection as detected by RPA accumulation at laser damage in G1 (CyclinA-negative) cells. Experiments were performed as in Figure 5E with indicated antibodies after 4 h post micro-irradiation. (**F**) RPA32 laser lines in CyclinA-negative cells without or with GFP-LANA (1–32a.a.) expression are indicated by yellow and green dotted lines respectively. Enlarged images from each category are shown. All cells have been laser damaged. (**G**) Quantification of F and G from either WT GFP-LANA (1–32a.a.) or mutant (Mut) GFP-LANA-8LRS10 expressing cells. Laser damaged CylclinA negative cells positive for LANA expression were scored for RPA laser line formation. Graph represents data obtained from >50 cells from two independent experiments. Error bars = SEM.

53BP1 functions in DNA double-strand break repair by both promoting NHEJ and inhibiting HR (reviewed in [38]). 53BP1 recruits the DDR factor RIF1 to DNA damage sites where it inhibits DNA end-resection and acts as the main effector of 53BP1-dependent NHEJ [39]–[43]. Consistent with GFP-LANA inhibiting RNF168-dependent 53BP1 recruitment, we also observed reduced RIF1 accumulation at IRIF in GFP-LANA expressing cells (Figure 6A). RNF168 is also required for the recruitment of the HR factor BRCA1 to DNA damage sites [23], [37]. Interestingly, GFP-LANA also impaired BRCA1 IRIF in S/G2 cells (Figure 6B; S/G2 cells were identified by CyclinA positive staining). Quantification of IRIF in GFP-LANA expressing cells revealed a greater than 50% reduction in cells with greater than 10 foci for either RIF1 or BRCA1 (Figure 6C, D). The ability of GFP-LANA to impair IRIF of DDR factors appears to be dependent on expression levels. We observed that high LANA expressing cells displayed a greater reduction in DDR factor recruitment compared to low LANA expressing cells, which explains the incomplete inhibition of DDR factor recruitment to DNA damage sites by GFP-LANA (Figure 5E, 6B). 53BP1 also inhibits DNA-end resection in G1 to block HR and promote NHEJ [39], [41], [42], [44]. Since expression of GFP-LANA impaired 53BP1 foci formation at DNA damage sites, we analyzed whether these cells exhibited functional inhibition of 53BP1 by monitoring DNA end-resection in G1 cells. RPA is recruited to, and binds, resected DNA, which is normally restricted to CyclinA-positive S/G2 cells. As expected, in control cells that do not express GFP-LANA or cells expressing mutant GFP-LANA-8LRS10, RPA foci at laser damage were virtually undetectable using our experimental conditions (Figure 6E, F, quantified in G). Interestingly, GFP-LANA expressing cells readily formed RPA foci at laser damage in CyclinA-negative G1 cells (Figure 6E, F, quantified in G). Thus, GFP-LANA expression resulted in DNA end-resection in G1 cells, which supports our previous results showing impaired 53BP1 recruitment to DNA damage by GFP-LANA. Taken together, these results are consistent with a role for the nucleosome acidic patch in promoting both 53BP1 and BRCA1 DDR pathways by mediating RNF168-dependent DNA damage signaling *in vivo*.

**Figure 7:**
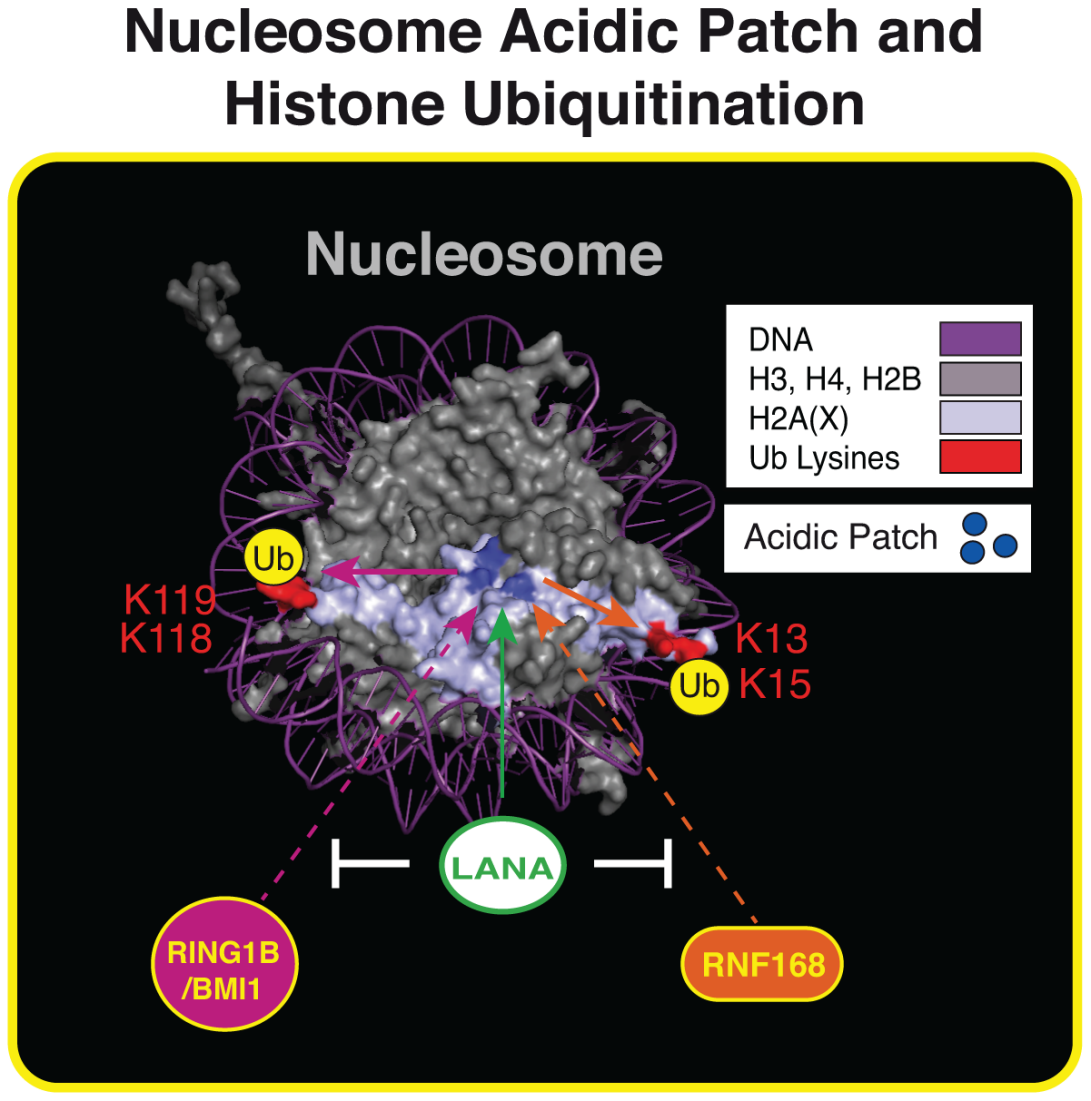
The nucleosome acidic patch and histone ubiquitination. Summary of our results within the context of the nucleosome structure. The acidic patch is required for RNF168- and RING1B/BMI1-dependent histone ubiquitination and LANA inhibits these processes. See text for details. Nucleosome structure was created in Pymol as previously described [20].

In summary, our results support a model whereby RNF168 and RING1B/BMI1 require the nucleosome acidic patch on H2AX/H2A to target these histones on site-specific lysines and that GFP-LANA can inhibit these processes (Figure 7). By overcoming the limitations of mutating the acidic patch of both H2A and H2AX through the expression of GFP-LANA, we have determined that the nucleosome acidic patch functions *in vivo* to promote RNF168-dependent DNA damage signaling. We have also created a novel tool that has the ability to silence DNA damage signaling at the level of RNF168 as well as inhibit RING1B/BMI1-dependent H2AX/H2Aub *in vivo*, which could be useful for studying these ubiquitin-dependent processes in cells. Of note, some viruses inactive the DDR by ubiquitin-dependent degradation mechanisms that target DDR factors, including RNF168 [45], [46]. Our results suggest that viruses, including LANA expressing KSHV, could inactive the DDR through another means by interfering with the nucleosome acidic patch. This potential mechanism would inhibit H2A/H2AX ubiquitination and subsequent DNA damage responses whose inhibition can affect viral transcription and activation of latent viruses in mammalian cells [47]. Additionally, other nucleosome acidic patch binding factors, including RCC1 and HMGN2, could also potentially affect the DDR. RCC1 and HMGN2 have opposing effects on chromatin dynamics with RCC1 promoting condensation of DNA prior to mitosis and HMGN2 decompacting chromatin through interactions with linker histone H1 [48]. We envision that these factors could regulate the DDR in multiple ways including chromatin dynamics and/or competition with other nucleosome acidic patch interacting proteins including RNF168 and RING1B/BMI1. Additional studies are warranted to investigate the interplay between nucleosome interacting factors and the DDR.

To our knowledge, this study has identified the first nucleosome domain that participates in both H2A/H2AXub and the DDR in human cells. Most studies have focused on the role of histone modifications, including ubiquitination, in the DDR. Our findings provide evidence that the DDR engages the nucleosome acidic patch, which participates in promoting histone ubiquitinations that mediate DDR factor interactions with chromatin including 53BP1. Chromatin interaction motifs within both RNF168 and RING1B/BMI1 have been identified. For example, RNF168 contains multiple ubiquitin-binding domains that target RNF168 to chromatin [49] and the RING1B/BMI1 complex contains DNA binding activity that is critical for histone ubiquitination [50]. Similar to the bivalent reading of histone marks by 53BP1, our results suggest that the histone ubiquitin writers, RNF168 and RING1B/BMI1, utilize multivalent chromatin interactions, including the nucleosome acidic patch, to write their “histone code.”

## Methods

### Cell cultures

HEK293T and BOSC23 cells were cultured in RPMI 1640 medium supplemented with 10% fetal bovine serum and 1% penicillin/streptomycin. Human U2OS cells were grown in Dulbecco's modified Eagle's medium (DMEM) supplemented with 10% fetal bovine serum (FBS), 100 U/ml penicillin, 100 µg/ml streptomycin and 2 mM L-glutamine. WT and H2AX-deficient MCF10A cells were cultured in DMEM/F12 medium supplemented with 5% horse serum, EGF (20 ng/ml), hydrocortisone (0.5 mg/ml), cholera toxin (100 ng/ml), insulin (10 µg/ml) and 1% penicillin/streptomycin. Cells were kept at 37 C in a humidified incubator containing 5% CO_2_.

### Plasmids

Human H2AX cDNA was a generous gift from Dr. Michael Huen from The University of Hong Kong. WT and mutant human H2AX cDNAs were cloned into gateway compatible entry vector (pDONR201). The cDNAs were then subcloned into expression vectors harboring N-terminal SFB (S-protein/2×Flag/Streptavidin-binding peptide), 3×Flag, Myc epitope tag, GFP epitope tag or HA-Flag epitope tag as indicated. Bacterial expression vectors for core histones (human H2A, H2B, H3 and H4) in pET21 were previously described [51]. Human H2AX cDNA were cloned into gateway compatible entry vector (pDONR201) and subcloned into bacterial expression vector pDEST17 harboring N-terminal 6× His tag. Constructs containing the nucleosomal 601 sequence was a kind gift from Ilya Finkelstein (UT Austin). 5′-biotin tagged 601 nucleotide sequence was generated by PCR using the primer pairs: 5′(Btn) CTGGAGAATCCCGGTGCC (forward primer) and 5′ACAGGATGTATATATCTGACACG (reverse primer) to be used for reconstitution of nucleosomes. pET24b (+)-Bmi1-His_6_ (residues 1–108), pGEX-6P-1-RING1B (residues 1–116) and pPROEX HTa-RNF168 (residues1–113) were obtained as described [10]. Full length LANA cDNA was a gift from Chris Sullivan (UT Austin). The N-terminal 32 amino acids of LANA were PCR amplified with the following primers Forward: 5′-TTGTCGACATGGCGCCCCCGGGAATGCGCCTGA-3′; Reverse: 5′-TTTCTAGACTATCTTTCCGGAGACCTGTTTCG-3′ and cloned into eGFP-C1 (Clontech) vector using 5′-SalI and 3′-XbaI restriction sites to create GFP-LANA (1–32a.a.). Primers for mutating 8RLS10 to AAA of LANA have been described by a previous study and were used to mutate GFP-LANA (1–32a.a.) to GFP-LANA-8LRS10 [52]. All mutations were generated using site-directed mutagenesis following standard protocols. All plasmid inserts and mutations were confirmed by DNA sequencing.

### Antibodies

The primary antibodies used were as follows: mouse anti-FLAG antibody (Sigma-Aldrich; F1804), mouse anti-γH2AX (Cell Signaling; #9718) and (Millipore; #05-636), rabbit anti-53BP1 (Novus Biologicals; NB100-304), mouse anti-53BP1 (BD transduction laboratories; 612522), rabbit anti-MDC1 (Abcam; ab11169), goat anti-Rif1 (N-20) (Santa Cruz, sc-55979), rabbit anti-beta-tubulin (Abcam; ab6046), mouse anti-c-Myc (Santa Cruz; sc-40), rabbit anti-H2AX (Cell Signaling; #2595). Secondary antibodies for western blotting were as follows: anti-rabbit igG, HRP-linked (Cell Signaling; #7074), anti-mouse IgG, HRP-linked (Cell Signaling; #7076). Secondary antibodies for IF analysis from Invitrogen were as follows: Alexa Fluor 488 (Rabbit, A11034), Alexa Fluor 594 (Rabbit, A11037), Alexa Fluor 594 (Mouse, A11032), Alexa Fluor 594 (Goat, A11058), Alexa Fluor 647 (Rabbit, A21245) and Alexa Fluor 647 (Mouse, A21236). For experiments involving cell cycle analysis, the following antibodies were used; mouse anti-BRCA1 (D-9) (Santa Cruz; sc-6954), mouse anti-RPA32/RPA2 [9H8] (Abcam; ab2175), rabbit anti-Phospho-histone H3 (Ser10) (D2C8) (Cell Signaling, #3377) and rabbit anti-CyclinA (H-432) and (Santa Cruz; sc-751).

### Transfections and retrovirus infection

Mammalian expression (SFB-, Myc- and GFP-) vectors were transfected using lipofectamine 2000 according to manufacturer's instruction and HA-Flag-retroviral expression constructs were co-transfected with pCL-ampho in BOSC23 cells using Lipofectamine 2000 (Invitrogen) according to the manufacturer's instruction. Viruses were harvested and filtered at 48 h and 72 h after transfection. MCF10A H2AX^−/−^ cells were transduced by virus containing medium and selected by puromycin (2 µg/ml). The GFP-LANA (1–32a.a) or GFP-LANA-8LRS10 constructs were transfected into the U2OS cells using HilyMax (Dojindo) according to the manufacturer's instruction. After 24 h post-transfection, cells were treated with 2 Gy IR and processed 2 h post-treatment. A Faxitron X-ray machine (Faxitron X-ray Corporation) was used for gamma irradiation (IR).

### Western blotting analysis

Mammalian cells were lysed in NETN (150 mM NaCl, 1 mM EDTA, 10 mM Tris-Cl, pH 8.0, 0,5% Nonidet P-40 (v/v) containing protease inhibitors. Samples were separated by SDS-PAGE in sample loading buffer, transferred to PVDF membranes, incubated overnight in primary antibodies as indicated, followed by 1 h of incubation in HRP-conjugated secondary antibodies. Western blots were detected by standard chemiluminescence (GE Healthcare Amersham ECL prime) using a Bio-Rad Molecular Imager ChemiDoc XRS+ system.

### Laser micro-irradiation

U2OS cells were plated on glass-bottomed dishes (Willco Wells). Post 8 h of transfection with GFP-LANA (1–32a.a), cells were pre-sensitized with 10 µM of 5-bromo-2′deoxyuridine (BrdU) in normal DMEM medium for 20 h. Laser micro-irradiation was carried out with a Fluoview 1000 confocal microscope (Olympus). Laser setting and protocols were as previous described [53]. After incubation with the indicated time points, cells were fixed and analyzed by immunofluorescence and microscopic imaging as described below. For quantification, >50 cells were scored for all conditions from at least two independent experiments.

### Immunofluorescence (IF) and confocal microscopy

U2OS, HEK293T and MCF10A cells were grown on poly-L-lysine Cellware 12 mm round coverslips (BD Biosciences). After the indicated treatments in HEK293T or U2OS cells, samples were treated and processed for IF as previously described [54].For MCF10A cells, cells were pre-extracted by incubating coverslips in CSK buffer (10 mM PIPES, pH 6.8, 100 mM NaCl, 300 mM sucrose, 3 mM MgCl_2_, 1 mM EGTA, 0.5% (v/v) Triton X-100) for 10 min on ice before fixing followed by IF analysis as previously described [20]. Cells were imaged using an inverted Fluoview 1000 confocal microscope (Olympus) and Z-stacked images were analyzed with Fluoview 3.1 software. For IRIF quantification, >100 cells were counted for all conditions. Data was analyzed in Prism and graphs were plotted from data obtained from two or three independent experiments as indicated.

### FACS analysis

U2OS cells were transfected with either GFP-LANA, Myc-LANA or a control vector for 8 h. 24 h after transfection, cells were harvested and fixed for 24 h with 80% ethanol. The fixed samples were then washed three times with 1× PBS containing 1% FCS and incubated with phospho-histone H3 (S10) primary antibody for 2 h followed by incubation of goat anti-rabbit secondary antibody for 2 h at room temperature for the mitotic index assay. For DNA content analysis, the cells were washed and stained with propidium iodide and processed on a BD Accuri C6 flow cytometer (BD Biosciences) for cell cycle analysis.

### *In vitro* assays

#### Nucleosome core particle (NCP) reconstitution

Recombinant human histones were expressed in *E.coli* BL21 (DE3)/RIL cells from pET21 vectors and extracted from inclusion bodies as described [55]–[57]. All histones were purified under denaturing conditions on 5 ml HiTrap Q and HiTrap SP cation exchange columns (GE Healthcare). Peak fractions were confirmed using SDS-PAGE and fractions containing pure histones were pooled and dialyzed extensively into 10 mM Tris-HCl (pH 8.0) before lyophilization. Octamers were refolded from purified histones by mixing the four histones in equimolar ratios (10% more of H2A/H2B relative to H3/H4), followed by dialysis into 2M NaCl and then purified on a Superdex 200 (16/60) size exclusion column (GE Healthcare). NCPs were reconstituted by salt deposition as described [57]. The 147 bp ‘601’ DNA was biotinylated and used to wrap the mononucleosomes. NCP formations were confirmed on 6% native TBE gels by gel mobility shift assays.

#### Protein purifications

E3 Ub ligase enzymes used in our *in vitro* Ub assays were as follows; the His_6_ tagged RNF168 (1–113) construct was expressed in Rosetta 2 (DE3) pLysS cells and purified over a Ni-NTA column (Qiagen) and stored in elution buffer (50 mM NaH_2_PO_4_, 300 mM NaCl, 250 mM imidazole, pH 8.0) containing 10% glycerol. The pET24b(+)-Bmi1-His_6_ (1–108) and pGEX-6P-1-RING1B (1–116) expression plasmids were co-transformed in Rosetta 2 (DE3) pLysS cells and the proteins were purified as a complex as described [50].

#### *In vitro* Ub assay

Assays were performed essentially as described [10]. Briefly, 2.5 µg of recombinant mononucleosomes were incubated in a 50 µl reaction buffer containing 50 mM Tris-HCl, pH 7.5, 100 mM NaCl, 10 mM MgCl_2_, 1 µM ZnOAc, 1 mM DTT, 30 nM ubiquitin activating enzyme E1 (Boston Biochem), 1.5 µM ubiquitin conjugating enzyme UbcH5a (Boston Biochem), 4 µM RNF168 (1–113) or RING1B/BMI1 complex, 22 µM ubiquitin (Boston Biochem) and 3.33 mM ATP at 30°C for 4 h. The reaction was terminated by addition of SDS-PAGE loading buffer. Assays with free histones were carried out with 10 µM of H2A or H2AX and the reactions were incubated overnight at 30 C. The samples were boiled and loaded on 15% SDS-PAGE gels, transferred to a nitrocellulose membrane, probed using specific antibodies and detected as described above.

#### *In vitro* methylation assay

2.5 µg of recombinant mononucleosomes were incubated at 30°C for 2 h in a 50 µl reaction containing 50 mM Tris-HCl, pH 7.5, 100 mM NaCl, 10 mM MgCl_2_, 1 µM ZnOAc, 1 mM DTT, 0.1 mM S-adenosyl methionine (NEB), and 100 ng recombinant human Set8 (Active Motif). The reaction was terminated by addition of SDS-PAGE loading buffer. Western blots were probed for H2AX, H2A or H4K20me1 (Abcam).

**LANA competition assay.** *In vitro* Ub assays were set up as described above, except with 3, 10, 30, 50, 100 or 150 µM of the indicated peptide. The peptides were synthesized by Bio Basic: LANA (Biotin-Mini-PEG- MAPPGMRLRSGRSTGAPLTRGSY) and 8LRS10 (Biotin- Mini-PEG –MAPPGMRAAAGRSTGAPLTRGSY) and were based on data presented previously [32].

## Supporting Information

Figure S1 Mutation of the Acidic Patch Impairs Human H2AX/H2A Ubiquitination. (A and B) E92A mutation in the acidic patch reduces H2AX/H2Aub. WT or E92A H2AX/H2A constructs were transfected into HEK293T cells and analyzed by western blotting with the indicated antibodies. Arrows indicate ub forms. (SFB = S-tag, Flag epitope tag, and streptavidin-binding peptide tag; e = endogenous; tub = tubulin loading control). Molecular weights (kDa) are indicated on the left of each panel. HEK293T cells were used for all cellular assays.
(EPS)

Figure S2 H2AX-K118/119ub requires the Acidic Patch and is Unaffected by the Overexpression of RNF168. H2AX derivatives were transfected into HEK293T cells with or without Myc-RNF168 and either (−) or (+) IR treated with 20 Gy and allowed to recover for 6 h. Samples were harvested and analyzed by western blotting with the indicated antibodies. Arrows indicate H2AXub. (SFB = S-tag, Flag epitope tag, and streptavidin-binding peptide tag, e = endogenous).
(EPS)

Figure S3 The Acidic Patch Does Not Affect Set8 Methylation of NCPs. WT, H2AX-E92A (A) and H2A-E92A (B) NCPs were subjected to methylation assays with Set8. Samples were analyzed by western blotting with specific antibodies against H2AX, H2A and H4K20me1, a SET8-dependent methylation mark. The methylation reactions were performed for 2 h at 30 C. The different apparent molecular weight of WT H2A is due to a 6×His tag on WT H2A compared to untagged H2A-E92A. SAM = S-Adenosyl methionine.
(TIF)

Figure S4 IF Analysis of H2AX Derivatives Stably Expressed in MCF10A^−/−^ Cells. Cells analyzed in Figure 3C were probed with α-Flag to detect tagged-H2AX derivatives and DAPI identifies nuclear DNA. Cells were processed for IF as described in methods.
(TIF)

Figure S5 The Acidic Patch Interaction Region of LANA Inhibits H2Aub *in vitro*. Peptides derived from the KSHV acidic patch binding protein LANA compete with RING1B/BMI1-dependent H2Aub. *In vitro* Ub assays were performed (−) or (+) either LANA peptide or mutant LANA peptide (8LRS10) that does not interact with the nucleosome acidic patch. Assays were performed as in Figure 2 with increasing concentrations of peptides (µM) as indicated (2 h reactions).
(TIF)

Figure S6 *In vivo* expression of GFP-LANA (1–32a.a.) reduces 53BP1 IRIF in HEK293T cells. (A) HEK293T cells expressing GFP-LANA (1–32a.a) were analyzed as in Figure 5A. (B) Quantification of 53BP1 IRIF from A. Quantification and statistical analysis were performed as in Figure 5.
(EPS)

Figure S7 GFP-LANA (1–32 amino acids) expression does not alter the cell cycle in U2OS cells. (A) Cell cycle distributions of U2OS cells expressing GFP and GFP-LANA (1–32a.a) were analyzed by FACS and percentages of cells in G1, S, and G2/M are shown. GFP and GFP-LANA (1–32a.a) transfected cells were analyzed by western blotting with indicated antibodies. (B) Mitotic index from empty vector and Myc-LANA (1–32a.a) transfected cells were analyzed by anti-phospho-histone H3 (S10) staining and quantified by flow cytometry. Graph represents triplicate experiments. Error bars = SEM. (C–D) U2OS cells expressing GFP-LANA (1–32a.a) do not exhibit detectable changes in histone H3 (S10) phosporylation or nuclear DNA compaction. Untreated or IR-treated GFP-LANA (1–32a.a) transfected U2OS cells were analyzed as in Figure 5 with the indicated antibodies and DNA stain.
(EPS)

## References

[1] Kouzarides T (2007) Chromatin modifications and their function. Cell 128: 693–705. 17320507 10.1016/j.cell.2007.02.005

[2] Miller KM, Jackson SP (2012) Histone marks: repairing DNA breaks within the context of chromatin. Biochem Soc Trans 40: 370–376. 22435814 10.1042/BST20110747

[3] Rogakou EP, Pilch DR, Orr AH, Ivanova VS, Bonner WM (1998) DNA double-stranded breaks induce histone H2AX phosphorylation on serine 139. J Biol Chem 273: 5858–5868. 9488723 10.1074/jbc.273.10.5858

[4] Paull TT, Rogakou EP, Yamazaki V, Kirchgessner CU, Gellert M, et al (2000) A critical role for histone H2AX in recruitment of repair factors to nuclear foci after DNA damage. Curr Biol 10: 886–895. 10959836 10.1016/s0960-9822(00)00610-2

[5] Rogakou EP, Boon C, Redon C, Bonner WM (1999) Megabase chromatin domains involved in DNA double-strand breaks in vivo. J Cell Biol 146: 905–916. 10477747 10.1083/jcb.146.5.905 PMC2169482

[6] Jackson SP, Durocher D (2013) Regulation of DNA damage responses by ubiquitin and SUMO. Mol Cell 49: 795–807. 23416108 10.1016/j.molcel.2013.01.017

[7] Panier S, Durocher D (2013) Push back to respond better: regulatory inhibition of the DNA double-strand break response. Nat Rev Mol Cell Biol 14 10: 661–72. 24002223 10.1038/nrm3659

[8] Jackson SP, Bartek J (2009) The DNA-damage response in human biology and disease. Nature 461: 1071–1078. 19847258 10.1038/nature08467 PMC2906700

[9] Polo SE, Jackson SP (2011) Dynamics of DNA damage response proteins at DNA breaks: a focus on protein modifications. Genes Dev 25: 409–433. 21363960 10.1101/gad.2021311 PMC3049283

[10] Fradet-Turcotte A, Canny MD, Escribano-Diaz C, Orthwein A, Leung CC, et al (2013) 53BP1 is a reader of the DNA-damage-induced H2A Lys 15 ubiquitin mark. Nature 499: 50–54. 23760478 10.1038/nature12318 PMC3955401

[11] Mattiroli F, Vissers JH, van Dijk WJ, Ikpa P, Citterio E, et al (2012) RNF168 ubiquitinates K13–15 on H2A/H2AX to drive DNA damage signaling. Cell 150: 1182–1195. 22980979 10.1016/j.cell.2012.08.005

[12] Gatti M, Pinato S, Maspero E, Soffientini P, Polo S, et al (2012) A novel ubiquitin mark at the N-terminal tail of histone H2As targeted by RNF168 ubiquitin ligase. Cell Cycle 11: 2538–2544. 22713238 10.4161/cc.20919 PMC3404880

[13] Chagraoui J, Hebert J, Girard S, Sauvageau G (2011) An anticlastogenic function for the Polycomb Group gene Bmi1. Proc Natl Acad Sci U S A 108: 5284–5289. 21402923 10.1073/pnas.1014263108 PMC3069154

[14] Facchino S, Abdouh M, Chatoo W, Bernier G (2010) BMI1 confers radioresistance to normal and cancerous neural stem cells through recruitment of the DNA damage response machinery. J Neurosci 30: 10096–10111. 20668194 10.1523/JNEUROSCI.1634-10.2010 PMC6633363

[15] Ginjala V, Nacerddine K, Kulkarni A, Oza J, Hill SJ, et al (2011) BMI1 is recruited to DNA breaks and contributes to DNA damage-induced H2A ubiquitination and repair. Mol Cell Biol 31: 1972–1982. 21383063 10.1128/MCB.00981-10 PMC3133356

[16] Ismail IH, Andrin C, McDonald D, Hendzel MJ (2010) BMI1-mediated histone ubiquitylation promotes DNA double-strand break repair. J Cell Biol 191: 45–60. 20921134 10.1083/jcb.201003034 PMC2953429

[17] Fradet-Turcotte A, Canny MD, Escribano-Diaz C, Orthwein A, Leung CC, et al (2013) 53BP1 is a reader of the DNA-damage-induced H2A Lys 15 ubiquitin mark. Nature 499 7456: 50–4. 23760478 10.1038/nature12318 PMC3955401

[18] Sobhian B, Shao G, Lilli DR, Culhane AC, Moreau LA, et al (2007) RAP80 targets BRCA1 to specific ubiquitin structures at DNA damage sites. Science 316: 1198–1202. 17525341 10.1126/science.1139516 PMC2706583

[19] Lukas J, Lukas C, Bartek J (2011) More than just a focus: The chromatin response to DNA damage and its role in genome integrity maintenance. Nat Cell Biol 13: 1161–1169. 21968989 10.1038/ncb2344

[20] Chen WT, Alpert A, Leiter C, Gong F, Jackson SP, et al (2013) Systematic identification of functional residues in mammalian histone H2AX. Mol Cell Biol 33: 111–126. 23109425 10.1128/MCB.01024-12 PMC3536310

[21] Wang H, Wang L, Erdjument-Bromage H, Vidal M, Tempst P, et al (2004) Role of histone H2A ubiquitination in Polycomb silencing. Nature 431: 873–878. 15386022 10.1038/nature02985

[22] Gudjonsson T, Altmeyer M, Savic V, Toledo L, Dinant C, et al (2012) TRIP12 and UBR5 suppress spreading of chromatin ubiquitylation at damaged chromosomes. Cell 150: 697–709. 22884692 10.1016/j.cell.2012.06.039

[23] Doil C, Mailand N, Bekker-Jensen S, Menard P, Larsen DH, et al (2009) RNF168 binds and amplifies ubiquitin conjugates on damaged chromosomes to allow accumulation of repair proteins. Cell 136: 435–446. 19203579 10.1016/j.cell.2008.12.041

[24] Stucki M, Clapperton JA, Mohammad D, Yaffe MB, Smerdon SJ, et al (2005) MDC1 directly binds phosphorylated histone H2AX to regulate cellular responses to DNA double-strand breaks. Cell 123: 1213–1226. 16377563 10.1016/j.cell.2005.09.038

[25] Huen MS, Grant R, Manke I, Minn K, Yu X, et al (2007) RNF8 transduces the DNA-damage signal via histone ubiquitylation and checkpoint protein assembly. Cell 131: 901–914. 18001825 10.1016/j.cell.2007.09.041 PMC2149842

[26] Kolas NK, Chapman JR, Nakada S, Ylanko J, Chahwan R, et al (2007) Orchestration of the DNA-damage response by the RNF8 ubiquitin ligase. Science 318: 1637–1640. 18006705 10.1126/science.1150034 PMC2430610

[27] Mailand N, Bekker-Jensen S, Faustrup H, Melander F, Bartek J, et al (2007) RNF8 ubiquitylates histones at DNA double-strand breaks and promotes assembly of repair proteins. Cell 131: 887–900. 18001824 10.1016/j.cell.2007.09.040

[28] Fang J, Feng Q, Ketel CS, Wang H, Cao R, et al (2002) Purification and functional characterization of SET8, a nucleosomal histone H4-lysine 20-specific methyltransferase. Curr Biol 12: 1086–1099. 12121615 10.1016/s0960-9822(02)00924-7

[29] Ismail IH, McDonald D, Strickfaden H, Xu Z, Hendzel MJ (2013) A small molecule inhibitor of Polycomb repressive complex 1 inhibits ubiquitin signaling at DNA double-strand breaks. J Biol Chem 288 37: 26944–54. 23902761 10.1074/jbc.M113.461699 PMC3772243

[30] Pan MR, Peng G, Hung WC, Lin SY (2011) Monoubiquitination of H2AX protein regulates DNA damage response signaling. J Biol Chem 286: 28599–28607. 21676867 10.1074/jbc.M111.256297 PMC3151101

[31] Wu CY, Kang HY, Yang WL, Wu J, Jeong YS, et al (2011) Critical role of monoubiquitination of histone H2AX protein in histone H2AX phosphorylation and DNA damage response. J Biol Chem 286: 30806–30815. 21690091 10.1074/jbc.M111.257469 PMC3162441

[32] Barbera AJ, Chodaparambil JV, Kelley-Clarke B, Joukov V, Walter JC, et al (2006) The nucleosomal surface as a docking station for Kaposi's sarcoma herpesvirus LANA. Science 311: 856–861. 16469929 10.1126/science.1120541

[33] Kato H, van Ingen H, Zhou BR, Feng H, Bustin M, et al (2011) Architecture of the high mobility group nucleosomal protein 2-nucleosome complex as revealed by methyl-based NMR. Proc Natl Acad Sci U S A 108: 12283–12288. 21730181 10.1073/pnas.1105848108 PMC3145696

[34] Luger K, Mader AW, Richmond RK, Sargent DF, Richmond TJ (1997) Crystal structure of the nucleosome core particle at 2.8 A resolution. Nature 389: 251–260. 9305837 10.1038/38444

[35] Makde RD, England JR, Yennawar HP, Tan S (2010) Structure of RCC1 chromatin factor bound to the nucleosome core particle. Nature 467: 562–566. 20739938 10.1038/nature09321 PMC3168546

[36] Roussel L, Erard M, Cayrol C, Girard JP (2008) Molecular mimicry between IL-33 and KSHV for attachment to chromatin through the H2A-H2B acidic pocket. EMBO Rep 9: 1006–1012. 18688256 10.1038/embor.2008.145 PMC2572127

[37] Stewart GS, Panier S, Townsend K, Al-Hakim AK, Kolas NK, et al (2009) The RIDDLE syndrome protein mediates a ubiquitin-dependent signaling cascade at sites of DNA damage. Cell 136: 420–434. 19203578 10.1016/j.cell.2008.12.042

[38] Zimmermann M, de Lange T (2013) 53BP1: pro choice in DNA repair. Trends Cell Biol pii: S0962-8924(13)00155-4. 24094932 10.1016/j.tcb.2013.09.003 PMC3946699

[39] Chapman JR, Barral P, Vannier JB, Borel V, Steger M, et al (2013) RIF1 is essential for 53BP1-dependent nonhomologous end joining and suppression of DNA double-strand break resection. Mol Cell 49: 858–871. 23333305 10.1016/j.molcel.2013.01.002 PMC3594748

[40] Di Virgilio M, Callen E, Yamane A, Zhang W, Jankovic M, et al (2013) Rif1 prevents resection of DNA breaks and promotes immunoglobulin class switching. Science 339: 711–715. 23306439 10.1126/science.1230624 PMC3815530

[41] Escribano-Diaz C, Orthwein A, Fradet-Turcotte A, Xing M, Young JT, et al (2013) A cell cycle-dependent regulatory circuit composed of 53BP1-RIF1 and BRCA1-CtIP controls DNA repair pathway choice. Mol Cell 49: 872–883. 23333306 10.1016/j.molcel.2013.01.001

[42] Feng L, Fong KW, Wang J, Wang W, Chen J (2013) RIF1 counteracts BRCA1-mediated end resection during DNA repair. J Biol Chem 288: 11135–11143. 23486525 10.1074/jbc.M113.457440 PMC3630874

[43] Zimmermann M, Lottersberger F, Buonomo SB, Sfeir A, de Lange T (2013) 53BP1 regulates DSB repair using Rif1 to control 5′ end resection. Science 339: 700–704. 23306437 10.1126/science.1231573 PMC3664841

[44] Bunting SF, Callen E, Wong N, Chen HT, Polato F, et al (2010) 53BP1 inhibits homologous recombination in Brca1-deficient cells by blocking resection of DNA breaks. Cell 141: 243–254. 20362325 10.1016/j.cell.2010.03.012 PMC2857570

[45] Chaurushiya MS, Lilley CE, Aslanian A, Meisenhelder J, Scott DC, et al (2012) Viral E3 ubiquitin ligase-mediated degradation of a cellular E3: viral mimicry of a cellular phosphorylation mark targets the RNF8 FHA domain. Mol Cell 46: 79–90. 22405594 10.1016/j.molcel.2012.02.004 PMC3648639

[46] Lilley CE, Chaurushiya MS, Boutell C, Landry S, Suh J, et al (2010) A viral E3 ligase targets RNF8 and RNF168 to control histone ubiquitination and DNA damage responses. EMBO J 29: 943–955. 20075863 10.1038/emboj.2009.400 PMC2837166

[47] Weitzman MD, Lilley CE, Chaurushiya MS (2010) Genomes in conflict: maintaining genome integrity during virus infection. Annu Rev Microbiol 64: 61–81. 20690823 10.1146/annurev.micro.112408.134016

[48] Kalashnikova AA, Porter-Goff ME, Muthurajan UM, Luger K, Hansen JC (2013) The role of the nucleosome acidic patch in modulating higher order chromatin structure. J R Soc Interface 10: 20121022. 23446052 10.1098/rsif.2012.1022 PMC3627075

[49] Panier S, Ichijima Y, Fradet-Turcotte A, Leung CC, Kaustov L, et al (2012) Tandem protein interaction modules organize the ubiquitin-dependent response to DNA double-strand breaks. Mol Cell 47: 383–395. 22742833 10.1016/j.molcel.2012.05.045

[50] Bentley ML, Corn JE, Dong KC, Phung Q, Cheung TK, et al (2011) Recognition of UbcH5c and the nucleosome by the Bmi1/Ring1b ubiquitin ligase complex. EMBO J 30: 3285–3297. 21772249 10.1038/emboj.2011.243 PMC3160663

[51] Tjeertes JV, Miller KM, Jackson SP (2009) Screen for DNA-damage-responsive histone modifications identifies H3K9Ac and H3K56Ac in human cells. EMBO J 28: 1878–1889. 19407812 10.1038/emboj.2009.119 PMC2684025

[52] Barbera AJ, Ballestas ME, Kaye KM (2004) The Kaposi's sarcoma-associated herpesvirus latency-associated nuclear antigen 1 N terminus is essential for chromosome association, DNA replication, and episome persistence. J Virol 78: 294–301. 14671111 10.1128/JVI.78.1.294-301.2004 PMC303411

[53] Shee C, Cox BD, Gu F, Luengas EM, Joshi MC, et al (2013) Engineered proteins detect spontaneous DNA breakage in human and bacterial cells. Elife 2: e01222. 24171103 10.7554/eLife.01222 PMC3809393

[54] Miller KM, Tjeertes JV, Coates J, Legube G, Polo SE, et al (2010) Human HDAC1 and HDAC2 function in the DNA-damage response to promote DNA nonhomologous end-joining. Nat Struct Mol Biol 17: 1144–1151. 20802485 10.1038/nsmb.1899 PMC3018776

[55] Bartke T, Vermeulen M, Xhemalce B, Robson SC, Mann M, et al (2010) Nucleosome-interacting proteins regulated by DNA and histone methylation. Cell 143: 470–484. 21029866 10.1016/j.cell.2010.10.012 PMC3640253

[56] Dyer PN, Edayathumangalam RS, White CL, Bao Y, Chakravarthy S, et al (2004) Reconstitution of nucleosome core particles from recombinant histones and DNA. Methods Enzymol 375: 23–44. 14870657 10.1016/s0076-6879(03)75002-2

[57] Lee JY, Greene EC (2011) Assembly of recombinant nucleosomes on nanofabricated DNA curtains for single-molecule imaging. Methods Mol Biol 778: 243–258. 21809211 10.1007/978-1-61779-261-8_16

