# Supplementary figures and images for: Nucleosome Acidic Patch Promotes RNF168- and RING1B/BMI1-Dependent H2AX and H2A Ubiquitination and DNA Damage Signaling
Source: PLoS Genet. 2014 Mar 6;10(3):e1004178. doi: 10.1371/journal.pgen.1004178 (PMC3945288; doi:10.1371/journal.pgen.1004178)

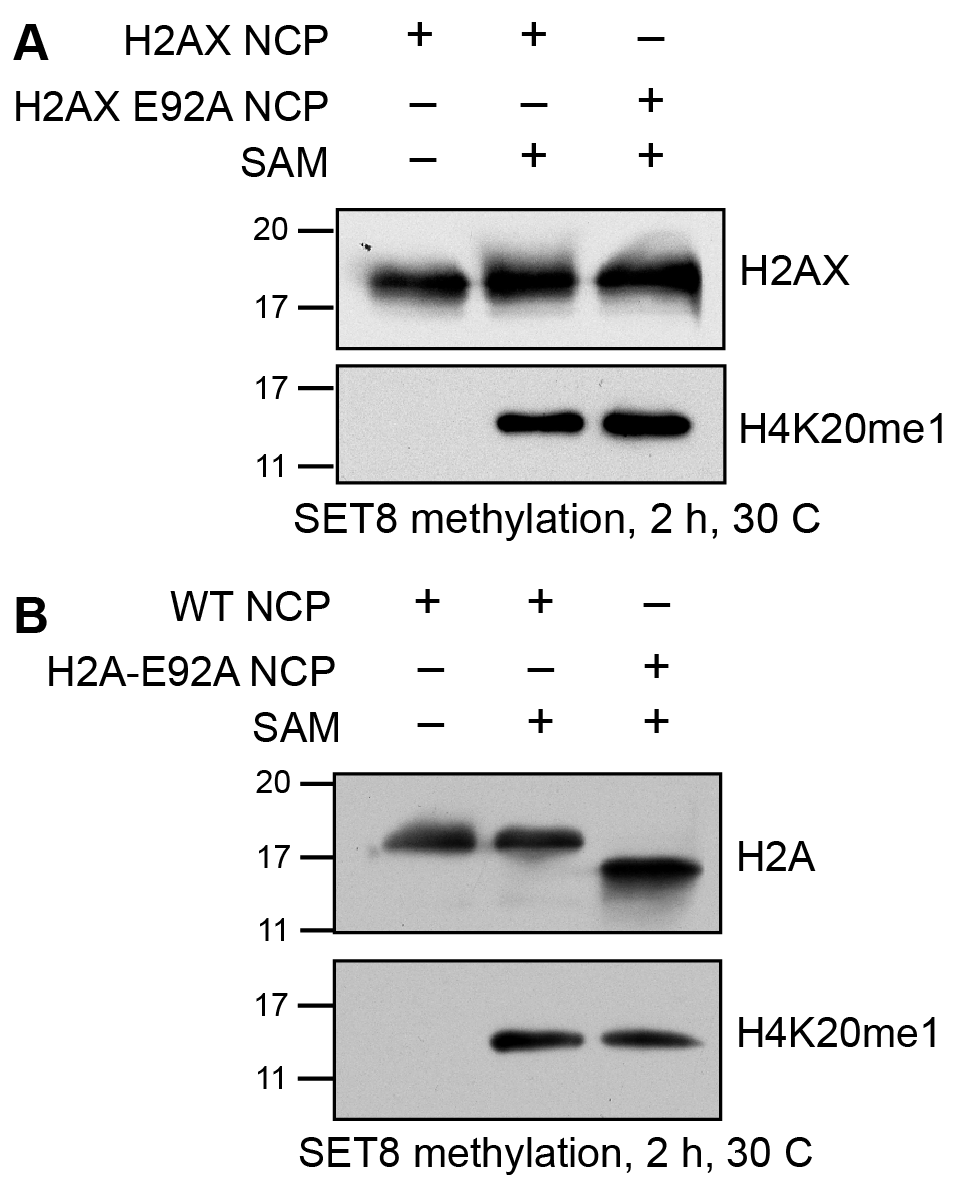

Supplement: Figure S3 — The Acidic Patch Does Not Affect Set8 Methylation of NCPs. WT, H2AX-E92A (A) and H2A-E92A (B) NCPs were subjected to methylation assays with Set8. Samples were analyzed by western blotting with specific antibodies against H2AX, H2A and H4K20me1, a SET8-dependent methylation mark. The methylation reactions were performed for 2 h at 30 C. The different apparent molecular weight of WT H2A is due to a 6×His tag on WT H2A compared to untagged H2A-E92A. SAM = S-Adenosyl methionine. (TIF) [file pgen.1004178.s003.tif]

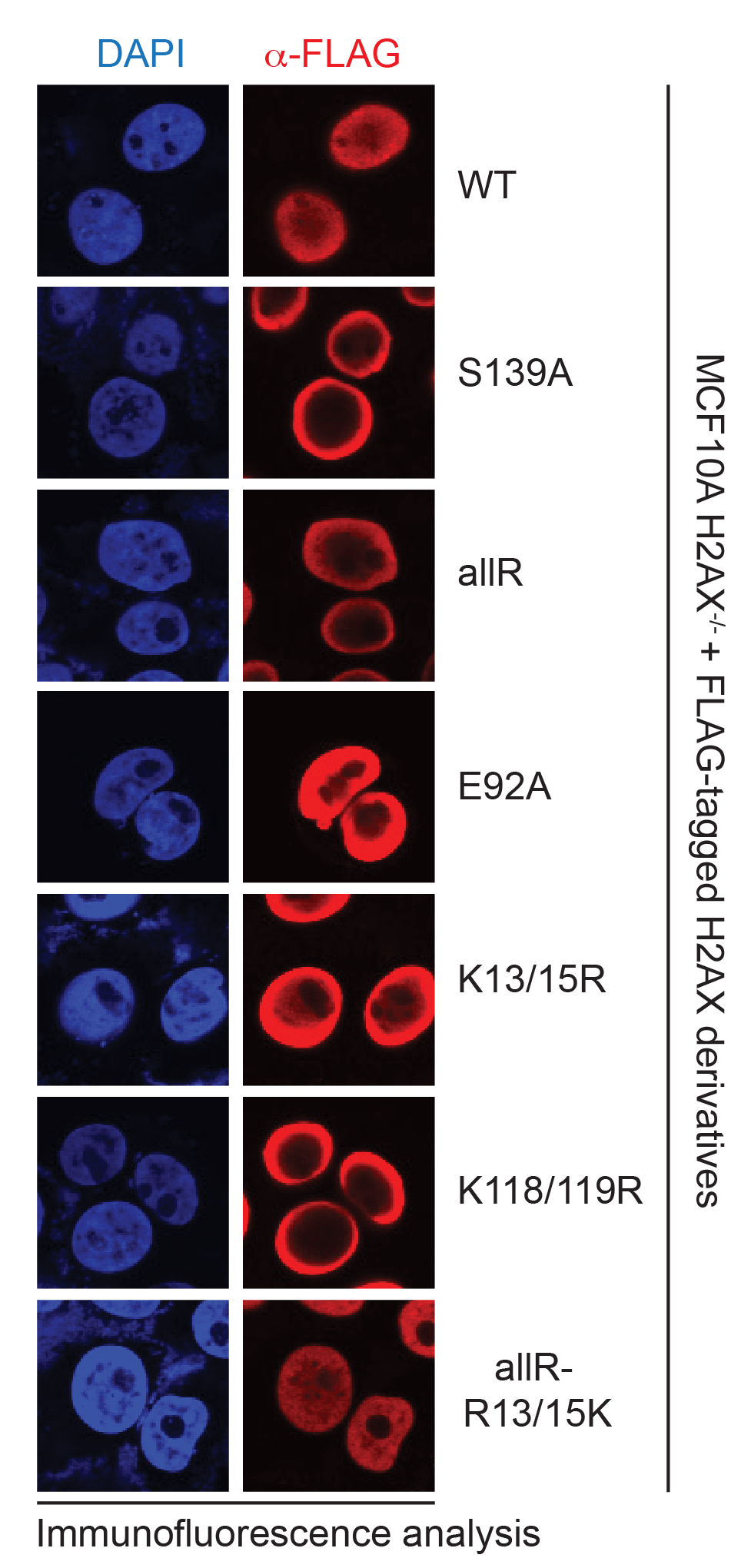

Supplement: Figure S4 — IF Analysis of H2AX Derivatives Stably Expressed in MCF10A−/− Cells. Cells analyzed in Figure 3C were probed with α-Flag to detect tagged-H2AX derivatives and DAPI identifies nuclear DNA. Cells were processed for IF as described in methods. (TIF) [file pgen.1004178.s004.tif]

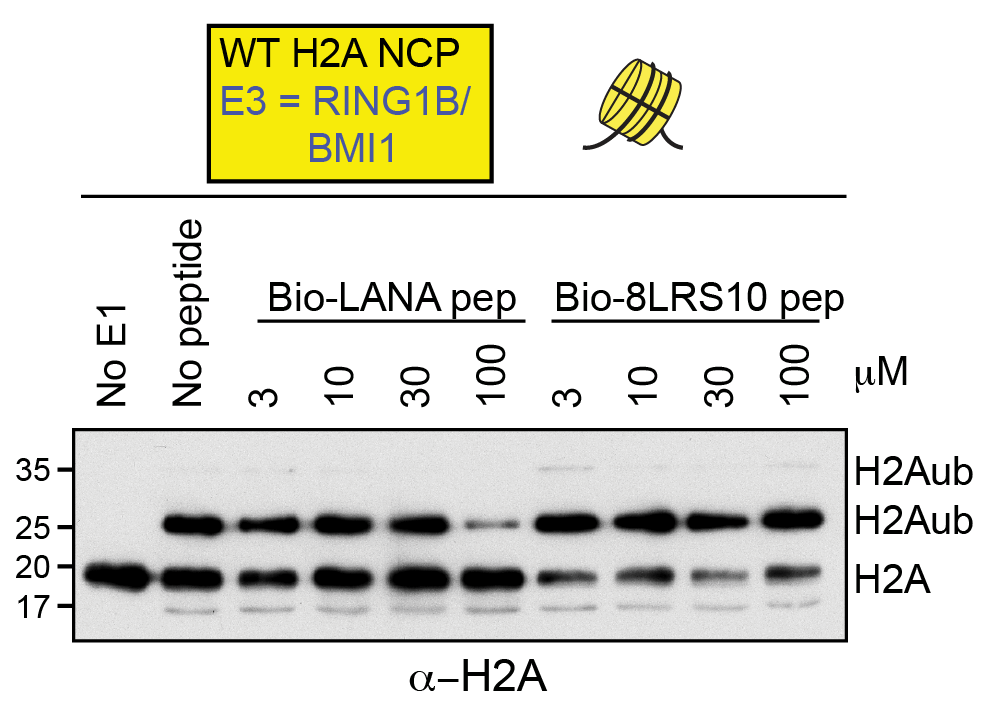

Supplement: Figure S5 — The Acidic Patch Interaction Region of LANA Inhibits H2Aub in vitro. Peptides derived from the KSHV acidic patch binding protein LANA compete with RING1B/BMI1-dependent H2Aub. In vitro Ub assays were performed (−) or (+) either LANA peptide or mutant LANA peptide (8LRS10) that does not interact with the nucleosome acidic patch. Assays were performed as in Figure 2 with increasing concentrations of peptides (µM) as indicated (2 h reactions). (TIF) [file pgen.1004178.s005.tif]
